# Dry Powder Formulation of Simvastatin Nanoparticles for Potential Application in Pulmonary Arterial Hypertension

**DOI:** 10.3390/pharmaceutics14050895

**Published:** 2022-04-20

**Authors:** Shalaleh Zendehdel Baher, Shadi Yaqoubi, Kofi Asare-Addo, Hamed Hamishehkar, Ali Nokhodchi

**Affiliations:** 1 Biotechnology Research Center, Students’ Research Committee and Faculty of Pharmacy, Tabriz University of Medical Sciences, Tabriz 51368, Iran; zendedel.baher@gmail.com; 2 Research Center for Integrative Medicine in Aging and Faculty of Pharmacy, Tabriz University of Medical Sciences, Tabriz 51368, Iran; shadi.yaqoubi@gmail.com; 3 Department of Pharmacy, School of Applied Sciences, University of Huddersfield, Huddersfield HD1 3DH, UK; k.asare-addo@hud.ac.uk; 4 Drug Applied Research Center, Tabriz University of Medical Sciences, Tabriz 51368, Iran; 5 Pharmaceutics Research Laboratory, School of Life Sciences, University of Sussex, Brighton BN1 9RH, UK

**Keywords:** dry powder inhaler, idiopathic pulmonary arterial hypertension, nanoparticle, simvastatin, pulmonary drug delivery

## Abstract

It has been hypothesized that simvastatin could be used to treat pulmonary arterial hypertension (PAH). This study is intended to formulate a simvastatin nanoparticle dry powder inhalation (DPI) formulation. Simvastatin nanoparticles were prepared via an emulsification and homogenization-extrusion method, followed by spray drying of the colloidal suspension of simvastatin nanoparticles containing mannitol to get it into a respirable size. Particle size distribution, morphology, and crystallinity of the fabricated nanoparticles of the obtained microparticles for DPI formulation were assessed by dynamic light scattering (DLS), scanning electron microscopy (SEM), and X-ray diffraction pattern (XRPD), respectively. Aerosolization performance of the DPI formulation was assessed by the Next Generation Impactor (NGI) equipped with an Aerolizer^®^. Simvastatin nanoparticles were around 100 nm with a very narrow size distribution (PDI = 0.105). The X-ray diffraction pattern revealed that the crystallinity of simvastatin was decreased by the spray drying procedure. Microscopic images displayed that gathered nanoparticles were in the suitable inhalable range and had the appropriate shape and surface properties for pulmonary delivery. Aerosolization assessment by the NGI indicated a suitable inhalation performance (fine particle fraction of 20%). In conclusion, the results confirmed that the spray drying technique for simvastatin can be optimized to obtain simvastatin aggregated nanoparticles without any coarse carrier to be used in DPI formulation for better deposition of the drug in the lungs for local treatment of PAH.

## 1. Introduction

Idiopathic pulmonary arterial hypertension (IPAH) is a rare and debilitating disease described by vascular proliferation and remodeling of the small pulmonary arteries with no apparent cause. This disease increases the pulmonary vascular resistance, augments afterwards in the right ventricle side and finally causes right heart failure [1]. The treatment of IPAH has developed quickly in the recent decade from the use of non-selective vasodilators, which specifically target pulmonary vasodilatation [2,3]. Statins, especially simvastatin, present a new approach to the treatment of IPAH [4,5,6]. Besides, apoptosis increases by decreasing proliferation of smooth muscle cells in blockage vascular lesions [7]. The oral bioavailability of simvastatin is around 5% due to first-pass metabolism. This can be increased by drug administration via the pulmonary route. In addition, the chance of drug interaction is high for simvastatin because of its hepatic metabolism by cytochrome P450, since the performance of this enzyme is influenced by a wide range of drugs and foods. Pulmonary drug delivery systems, particularly dry powder inhalers (DPI) have become one of the most popular drug delivery systems for reasons such as their capability in delivering a sufficient and necessary dose of a drug to the lung with minimum systemic side effects; escaping from liver first-pass effects, and providing a fast response since the drug is delivered directly to the desired zone of the lungs. Although, there are a few studies confirming the role of simvastatin in pulmonary hypertension therapy, there is no reported work on simvastatin formulation for pulmonary delivery. There are three clinically successful pulmonary inhalation dosage forms based on device classes, nebulizers (nebs), metered dose inhalers (MDI), and dry powder inhalers (DPI). Nebulizers don’t have any propellant and make liquid aerosols by means of an external power supply. Their use is limited to clinical settings. DPIs include respirable powdered drug or respirable powdered drug blended with a non-respirable carrier. DPIs are also very useful for poorly water-soluble drugs like simvastatin. Among the various types of pulmonary drug delivery systems, DPIs offer many advantages [8,9,10]. These include factors such as creating an opportunity to enhance physicochemical stability, modified pharmacokinetics, extended release profile especially in nano size, and direct delivery of the drug to the site of action in the lungs. Furthermore, they do not need cold chain storage and solubilization of the drug in a vehicle as in nebulizers [11,12]. However, unlike metered-dose inhalers (MDIs), DPIs do not have propellants, so the dispersion energy of the powder is usually obtained from the inhalation effort exerted by the patient [13].

Recently, nanotechnology has opened a new horizon for enhancing the therapeutic performance of medicines [14,15]. In this approach, lung drug delivery has many benefits [16] such as fast therapeutic action because of rapid dissolution in lung fluid, which is very crucial for hydrophobic drugs like simvastatin. The lung has two main clearance mechanisms (mucocilliary clearance and alveolar macrophage clearance) that prevent foreign particles arriving to the body and maintain the sterility of the lung; however, they have a negative effect on pulmonary drug delivery. Simvastatin nanoparticles are able to escape from the lungs’ natural clearance mechanisms since nanoparticles rapidly reach the epithelium and deposit in the alveolar zone and alveolar macrophages could engulf particles in sizes ranged between 1.5–3 µm. In addition, pulmonary drug delivery in nano size is useful for target delivery, sustained delivery and deep lung delivery of drugs [17]. On the other hand, drug particles with mean diameters of less than 0.5 µm were pulled out with exhalation. To overcome this challenge, drug nanoparticles are often gathered in loose agglomerates in the respirable size (1–5 µm) named Trojan particles or strawberry-like particles [18,19]. Based on the above explanation, the DPI formulation of simvastatin in the nano-size can offer many merits for enhanced IPAH treatment which has not reported before. To the best of our knowledge, there is no research on attempting to design inhalable simvastatin nanoparticles and spray drying of these nanoparticles containing mannitol to achieve respirable size for lung delivery. Therefore, the goal of this research is to formulate an optimized dry powder carrier-free inhalable formulation of simvastatin using the spray drying method with the capability to be deposited in the lungs.

## 2. Materials and Methods

### 2.1. Materials

Simvastatin was supplied from Aria Ltd. (Tehran, Iran). Chloroform, acetonitrile, and methanol were purchased from Duksan Pure Chemicals (Ansan, Korea). Soybean phosphatidylcholine (SPC, purity >99%) was acquired from Lipoid GmbH (Ludwigshafen, Germany). D-Mannitol and ethanol were obtained from Fluka (Steinheim, Germany) and JATA (Iran) companies respectively. Tween^®^ 80, span^®^ 80, polyvinyl alcohol (PVA), and polyethylene glycol (PEG) 400 were acquired from Sigma-Aldrich (St Louis, MO, USA).

### 2.2. Preparation of Simvastatin Nanoparticles

In the preliminary studies, two preparation approaches, namely solvent displacement and emulsification techniques, were employed. In the case of the solvent displacement method, three water-miscible organic solvents namely ethanol, methanol, and acetonitrile were used. In the solvent displacement technique, simvastatin was dissolved in an organic solvent (see Table 1) and then the resultant organic phase was gradually added (at a rate of 0.4 mL/min using a syringe pump) to the aqueous phase (see Table 1) while homogenization (20,000 rpm) was taking place (Silent Crusher M, Heidolph, Germany). Although, this method using water miscible solvents is an effective means of particle preparation, it was unable to provide sub-100 nm particles. Therefore, the authors shifted to an emulsification approach using a water-immiscible solvent. In the emulsification method, various emulsifiers including Tween^®^ 80, span^®^ 80, PVA, PEG 400, lecithin, and poloxamer were used. In this method, the aqueous phase was gradually added to the water-immiscible organic phase under homogenization at 20,000 rpm. Various solvents, such as dichloromethane, chloroform, carbon tetrachloride, hexane, etc., can be used. The ideal solvent should have a low boiling point for its complete removal from the formulation after the nanoemulsion preparation and at least some degree of polar characteristics for proper emulsification. Among the above-mentioned organic solvents, chloroform seemed to have these merits and therefore was used. To reduce the size and the distribution of emulsion droplets, the optimum formulation (No. 13) prepared from the emulsification method was extruded (Azerbaijan composite technologists, Tabriz, Iran) three times through a 50 nm polycarbonate filter (Wattman, Kent, UK). Finally, the chloroform was evaporated using a rotary evaporator (TAT 94-1093, Teif AzarTeb, Tehran, Iran) to provide simvastatin nano-dispersion. The details of the formulations made in both methods are summarized in Table 1. Preliminary results showed that the solvent displacement method was unable to produce particle sizes in the nano range.

### 2.3. Morphology and Size Analyses of Nanoparticles

The mean particle size of the nanoparticles in the size range of sub 500 nm was determined by laser dynamic light scattering (Zetasizer, Malvern, UK). The study was performed at a temperature of 25 °C for the samples after dilution with filtered water (0.22 μm filters). The size, size distribution, and polydispersity index (PDI) value for the optimized formulation were also reported. The particle size of particles larger than 500 nm were measured with a laser diffraction particle size analyzer (SALD-1100, Shimadzu, Japan). The average particle size was expressed as the volume mean diameter. Span is a measure of the width of the size distribution.

$$\mathrm{Span}=\frac{D\left(v,90\right)-\;D\left(v,10\right)\;}{D\left(v,50\right)}$$

where D(v, 90), D(v, 10) and D(v, 50) are the equivalent volume diameters at 90, 10, and 50% cumulative volume, respectively.

To evaluate the morphology and the surface of the obtained particles, a scanning electron microscope (SEM, MIRA3, TESCAN instrument, Brno, Czech Republic) was employed. After gold coating the samples (samples mounted on a metal stub with double-sided adhesive tape before coating) under vacuum in an argon atmosphere, the images of the particles were obtained by a direct current sputter technique (Emitechk450X, Lewes, UK) [20].

### 2.4. Preparation of Aggregated Simvastatin Nanoparticles for DPI Formulation 

Mannitol (150 mg) was dissolved in nano-simvastatin aqueous dispersion (formulation 13 in Table 1) and spray dried under different conditions using a mini spray dryer (DSD-02, DorsaTech, Karaj, Iran) with an inlet temperature of 140 ± 5 °C, aspiration of 75% and spray flow of 8–10 mL/min After the spray drying of the prepared simvastatin solutions, the deposited particles in the cyclone of the spray dryer were collected and kept in a firmly closed container wrapped with aluminum foil and kept desiccated over silica gel at room temperature until required for extra studies.

### 2.5. Solid-State Analysis of Spray-Dried Particles

In order to explore any changes in the crystallinity of simvastatin before and after spray drying, an X-ray diffractometer (Siemens D500, Munich, Germany) was employed. Diffractograms were performed at a scanning speed of 2°/min and a chart speed of 0.6°/min.

### 2.6. Powder Flow Study

As the flowability of particles is important in DPI formulation design, the flow of the obtained particles was tested by the angle of repose technique according to a standard protocol as stated in General Chapter <1174> Powder flow in United States Pharmacopeia (USP) [21]. The simplest method for the determination of the angle of repose is the “poured” angle. In brief, a static heap of powder, when only gravity is acting upon it, will tend to form a conical mound. The sides of the heap formed in this way make an angle with the horizontal which is known as the cone angle of repose. The value of the angle of repose will be high if the powder is cohesive and low if the powder is non-cohesive. The angle of repose, which is an indication of the ability of powders to flow was calculated for plain simvastatin and spray dried simvastatin powders [22].

### 2.7. Solubility Studies

The solubility of plain simvastatin and spray-dried simvastatin particles was determined by adding an excess amount of plain simvastatin and spray-dried simvastatin, respectively, in 2 mL of artificial lung fluid (Gamble’s solution) adjusted at pH 7.4 to 7.8 (827 pH lab, Metrohm, Herisau, Switzerland) in screw-capped vials according to Table 2 [23]. The prepared mixtures were vortexed and then placed in a bath shaker with a constant temperature of 25 °C until equilibrium was reached (preliminary results showed that 48 h was sufficient to reach equilibrium). After 48 h, the samples were centrifuged at 12,000 rpm for 15 min (PIT320, Pole Ideal Tajhiz, Tehran, Iran). The supernatant layer was removed and was diluted with methanol, after which the concentration of simvastatin was determined by UV-spectrophotometry (Ultrospec 2000, Pharmacia Biotech, Cambridge, UK) at the wavelength of 238 nm.

### 2.8. Content Uniformity Analysis

Five samples (2 mg of each) of the prepared microparticles were dissolved in 1.5 mL of methanol and were shaken for 24 h to ensure the complete dissolution of the drug. After centrifuging at 12,000 rpm, the supernatant was separated and analyzed by the developed HPLC method to check the content uniformity of prepared spray-dried powder. Quantitative analysis of simvastatin was performed using an HPLC system (Knauer, Berlin, Germany), equipped with a C18 column (250 × 4.6 mm, 5 μm) with a precolumn. The mobile phase contained a mixture of acetonitrile and distilled water (70:30 respectively), and the flow rate was set up at 1.3 mL/min (the mobile phase was already degassed by a bath sonicator for 10 min). The drug was detected at a wavelength of 238 nm. The limit of detection (LOD) and limit of quantification (LOQ) were calculated to be 15 and 45 ng/mL, respectively.

### 2.9. Aerosolization Performance of DPI Formulation

The aerosolization efficiency of the produced simvastatin microparticles was studied by the next-generation impactor (NGI; Copley Scientific, Nottingham, UK) based on the USP monograph. A specific amount of powders (20 mg) was filled into gelatin capsules (size 3) and inserted into an Aerolizer^®^ (Novartis, Basel, Switzerland), a commercially available single-dose capsule-based DPI. The capsule is pierced inside the device before actuating into the NGI. The surface of the collection cups was coated with Tween^®^80 to avoid any bouncing of the particles. To this end, all eight collection cups of the NGI were soaked into an ethanolic solution of 1% Tween^®^80, followed by the evaporation of ethanol at room temperature. After inserting the cups in their places in the NGI, the device containing the formulation was attached to the induction port. The pump was run for 4 s at a flow rate of 60 L/min. The flow rate was calibrated by a flow meter (DFM 2000, Copley Scientific, Nottingham, UK) and fixed at 60 L/min. After aerosolization, all collection surfaces were washed with methanol to dissolve simvastatin. The amount of drug left in the capsules was also determined. The Copley Inhaler Testing Data Analysis Software (CITDAS V3.10, Copley Scientific, Nottingham, UK) was employed to calculate mass median aerodynamic diameter (MMAD) and geometric standard deviation (GSD) as expressed in the USP 32-NF 27 [24]. When the amount of drug in each stage of the NGI was determined, fine particle fraction (FPF) which is an indication of what percentage of drug particles are less than 5 μm was calculated [10]. FPF evaluates the fraction of particles that are assumed to deposit in the lung to induce a pharmacological effect [25].

## 3. Results and Discussion

### 3.1. Characterization of Simvastatin Nanocrystals and Aggregated Nanoparticles for DPI Formulation

Results of the particle size analysis showed that in the case of using the solvent displacement method, acetonitrile produced smaller particles in comparison with other solvents (e.g., methanol and ethanol) (Table 3 formulations 1–3). Thus, acetonitrile was selected to be used in formulations 3 to 8, where in these formulations the effect of the type of surfactant, their concentrations and the homogenization time on the particle size were investigated. The results showed that an increase in homogenization time resulted an increase in the particle size (compare formulations 5 and 6 in Table 3). Interestingly, when the temperature was increased from 25 to 40 °C in the solvent displacement method, at a constant homogenization time of 10 min, the particle size reduced from 1.12 µm to 0.941 µm (formulations 4 and 5 in Table 3). The reduction in the particle size of simvastatin was more pronounced when the temperature was kept at 40 °C but homogenization time was reduced from 10 to 5 min (note: formulation 6 was extruded). In addition, when lecithin as surfactant was replaced by Span (as the inner surfactant) and Poloxamer (as outer surfactant), the size of particles dramatically increased from 1.7 µm to 10.99 µm (compare formulations 3 and 7). As the goal was to achieve simvastatin nanoparticles, an attempt was made to replace the solvent displacement method with the emulsification method (formulations 8 to 13).

In the emulsification technique, the effect of various surfactants namely PVA, PEG, lecithin, Tween, and Span 80 were investigated. The results showed that the effect of lecithin (formulations 12 and 13) on particle size reduction was more noticeable compared to other surfactants (Table 3). Furthermore, the formulation subjected to extrusion (formulation 13) reduced particle size to around 100 nm with very narrow size distribution (PDI = 0.1) (formulation 13). This formulation was used for further analysis in the current research.

**Figure 1:**
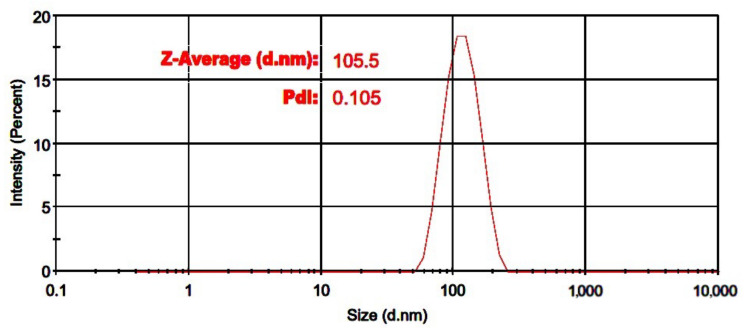
The size of distribution reported by intensity for the optimized formulation (formulation 13, Table 1).

The particle size distribution for the optimized formulation (formulation 13) is depicted in Figure 1. PDI value is important as it shows the uniformity of particles in size; where a low PDI value is desirable as it indicates a narrow particle size distribution. It has been proven that a PDI value > 0.2 is an indication of monodispersed particles [26]. Formulation 13 showed a PDI value of 0.1 which indicates this formulation contained monodispersed particles.

**Figure 2:**
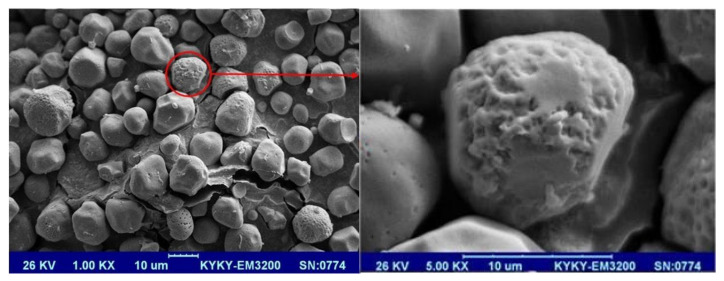
SEM images of the optimized formulation (formulation 13, Table 1) after spray drying.

DPI formulations were prepared with a spray drying method. Generally, particle size less than 1 µm can come out of the lungs during the exhalation due to their smaller size and low inertia. In order to keep these smaller particles in nano sized range, the particles should have the ability to aggregate to form particles in the respirable size of range 1–5 µm. This can happen spontaneously as nanoparticles tend to grow due to Ostwald ripening and also can become aggregated due to the high surface area of nanoparticles. Researchers have used spray drying to make nanoparticles into inhalable micro aggregates containing mannitol as a matrix [13]. Three different methods for spray drying were employed to obtain particles for formulation 13 with the conditions listed in Table 4 deemed the best method in obtaining the highest yield (Table 4). Thus, the spray drying method employed in the current study provided aggregated nanoparticles in the respirable range (<5 µm) (Figure 2) with an appropriate yield (65.8%).

To determine the microstructure, morphology, and particle size after spray drying, SEM was conducted and the images of the obtained particles (formulation 13) are shown in Figure 2. SEM images showed almost round shape particles with a mixture of smooth or some-times rough surfaces probably due to the presence of simvastatin nanoparticles in the surface of particles which provided some pores at the surfaces of the particles which were reported to be an appropriate particle design for pulmonary drug delivery [27,28]. The prepared spray dried formulation gave an angle of repose of around 33° which is an indication of good flow. The angle of repose for the unprocessed simvastatin was 37° which is considered fair flow. The angle of repose is an important parameter in DPI formulation as it has been reported that flow properties play a key role in aerosol performance of DPIs [29]. To ensure the simvastatin uniformity in final spray dried formulation, powder uniformity was assessed and found that the coefficient of variation (CV) of simvastatin was 5.16%. The CV percent of less than 6% is an indication of a good uniformity.

**Figure 3:**
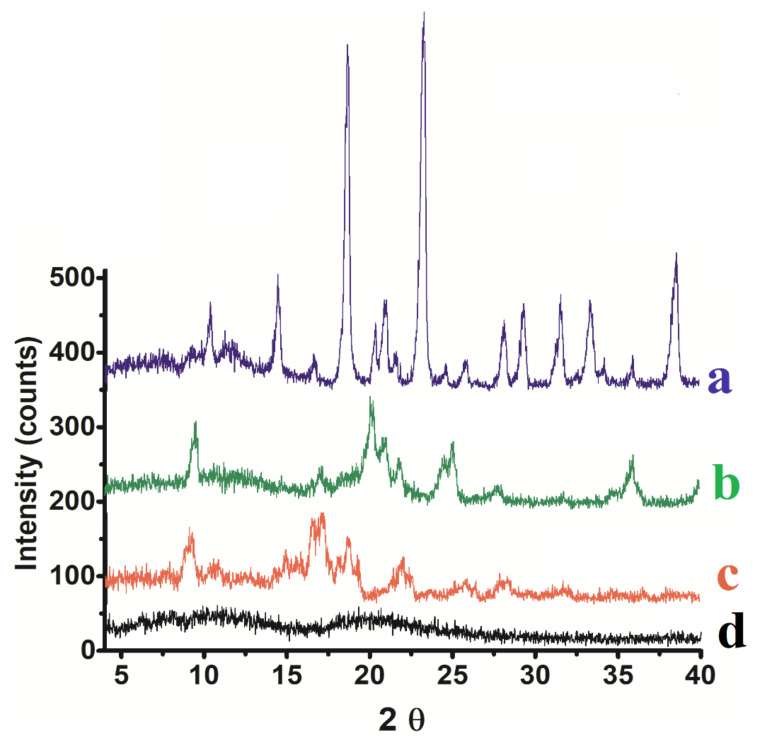
X-ray Diffraction pattern of mannitol (**a**), the spray dried optimized nano simvastatin (formulation 13, Table 3) (**b**), raw simvastatin (**c**), and lecithin (**d**).

XRPD analyses were performed on the carrier particles in order to evaluate possible changes in the crystallinity after spray drying. Figure 3 shows the XRPD of simvastatin powder before and after spray drying. The results indicated that the spray dried simvastatin seems to be slightly less crystalline than the original simvastatin as the intensity of peaks at 17 2θ was much lower than the untreated simvastatin. Peak at 9 2θ is almost unchanged, but mannitol crystallinity decreased dramatically which was obvious from XRD of spray dried nano-simvastatin formulation where many of sharp XRD peaks for mannitol disappeared. This could be due to a rapid arrangement of mannitol and simvastatin molecules during spray drying which can aid the dissolution of simvastatin in the lung for rapid and enhanced therapeutic performance [30].

### 3.2. Solubility Study

The solubility results of formulation 13 showed that the spray dried simvastatin (nanoparticles) exhibited higher solubility compared to unprocessed simvastatin powder. The solubility of simvastatin was 0.65 μg/mL, whereas the solubility value for simvastatin nanoparticles was 1.27 μg/mL which was an almost two-fold increase in the solubility in comparison with unprocessed simvastatin powder. The increase in the solubility of spray dried simvastatin could be due to a significant size reduction of simvastatin (around 100 nm) and a decrease in the crystallinity of simvastatin particles. Improvements in the solubility of hydrophobic drugs is extremely important in the design of DPI formulations as fast dissolution in the lungs can reduce premature mucociliary clearance, particularly in the case of hydrophobic drugs and improves the dissolution and pharmacokinetic profiles of the drugs. Furthermore, it has been reported that an enhancement in the solubility could be due to the decline in particle size and enhanced the wettability [31].

### 3.3. Aerosolization Performance of DPI Formulation

**Figure 4:**
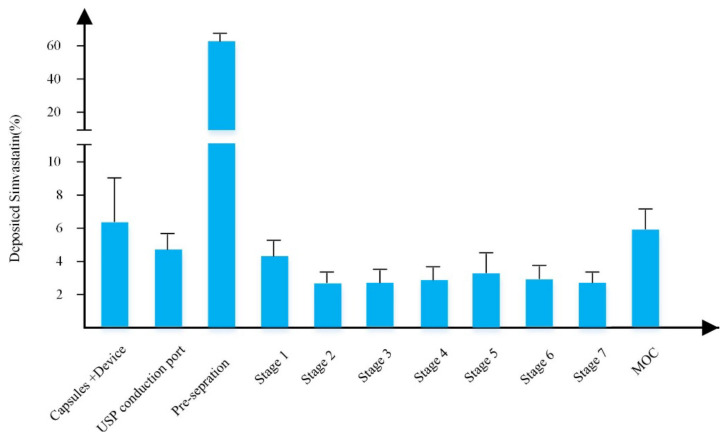
Aerosolisation performance of the optimized spray dried simvastatin in NGI (Data presented as mean ± SD, *n* = 3).

The SEM images shown in Figure 2 showed that the spray dried particles were in respirable size (1–5 µm). The spray dried simvastatin formulation was actuated into the NGI and the deposition results are shown in Figure 4. The results showed that although around 60% of the drug was deposited in the pre-separator, the amount of simvastatin deposited in the lower stages of the NGI was good enough for the delivery of simvastatin to the lungs. The results also showed that the value of FPF was 19.75 ± 2.45% with the mass median aerodynamic diameter (MMAD) of 1.33 ± 0.18 µm which makes these nanoparticles ideal for the delivery of drugs to the lungs. This research thus paves the way for the future effective therapy of IPAH for simvastatin via the pulmonary route [32,33].

## 4. Conclusions

In conclusion, this recent study focused on the development of simvastatin powders with an acceptable pulmonary deposition which can be highly affected by the physicochemical characteristics of drugs. One of the approaches to engineering drug particles for efficient delivery to the lungs is the use of spray drying technology. This current research showed that simvastatin particles without a carrier can be modified via emulsification, homogenization, and extrusion followed by optimizing spray drying conditions to produce loose aggregates of respirable size. The data showed that the loose aggregates contained simvastatin nanoparticles which can successfully be delivered to the lung without any carriers.

## Figures and Tables

**Table 1:** Composition and conditions used to make the different spray-dried formulations of simvastatin nanoparticles.

Formulation No.	Type and Amount of Internal Organic Phase (mL)	Amount of External Aqueous Phase (mL)	Type and Amount of Internal Phase Surfactant (mg)	Type and Amount of External Phase Surfactant (mg)	Homogenization Time (min)	Temperature (°C)	Extrude
1	Methanol-15	35	Lecithin (25) *	-	30	25	-
2	Ethanol-15	35	Lecithin (25)	-	30	25	-
3	Acetonitrile-15	20	Lecithin (25)	-	30	25	-
4	Acetonitrile-5	20	Lecithin (30)	-	10	25	-
5	Acetonitrile-5	20	Lecithin (30)	-	10	40	-
6	Acetonitrile-5	20	Lecithin (30)	-	5	40	√
7	Acetonitrile-15	35	Span (40)	Poloxamer (1.05)	20	25	-
8	Chloroform-5	15	Lecithin (80)	PEG (0.22)	15	25	√
9	Chloroform-2	10	Span (70)	Tween (700)	30	25	√
10	Chloroform-5	20	Span (80)	PVA (0.05)	30	25	√
11	Chloroform-5	15	Lecithin (140)	PVA (0.07)	30	25	√
12	Chloroform-5	10	Lecithin (105)	-	20	25	-
13	Chloroform-5	10	Lecithin (105)	-	20	25	√

* The values are the amount of surfactant.

**Table 2:** Composition of modified Gamble’s solution.

Materials	g/L
MgCl_2_·6H_2_O	0.212
NaCI	7.120
CaCI_2_·2H_2_O	0.029
Na_2_SO_4_	0.079
Na_2_HPO_4_	0.148
NaHCO_3_	1.950
Na_2_-tartrate 2H_2_O	0.180
Na_3_-citrate 2H_2_O	0.152
90% lactic acid	0.156
Glycine	0.118
Na-pyruvate	0.172
Formalin	1 mL

**Table 3:** Size characteristics of.

Sample No.	Size (μm)	PDI	Span (%)
1	3.36	-	2.870
2	2.51	-	0.873
3	1.70	-	1.260
4	1.12	-	0.578
5	0.941	-	1.849
6	0.640	-	0.778
7	10.99	-	1.830
8	1.021	-	1.605
9	1.339	-	1.6
10	1.110	-	1.252
11	1.135	-	1.543
12	0.380	0.219	-
13	0.103	0.105	-

PDI: Polydispersity index.

**Table 4:** Different conditions of the spray drying process used for obtaining high yield.

Parameters	T1	T2	T3
Nuzzle	6	6	6
Pump rate	3	1	3
Aspiration	65	75	75
Open-close time (S)	1–1	1–2	1–1
Q-FLOW	6	6	9
Temperature (C)	140°	140°	140°
Testing Time (min)	00:06′:15″	00:07′:15″	00:05′:47″
Yield (%)	38.2%	44.7%	65.8%

## Data Availability

Not applicable.
